# Medical Society of London, Feb. 22d, 1851

**Published:** 1851-07

**Authors:** 


### OBSTETRICS.

### Medical Society of London, Feb. 22d, 1851.

Dr. Tilt read a paper on *Diarrhœa as a hitherto Unnoticed Symptom of Menstruation, and on the use of Purgatives at the different Epochs of Menstruation.*

He premised that Friend and Brierre de Boismont not having given a description of catamenial diarrhœa in their works, he concluded that it was not generally known to the profession. He then inquired into the nature and frequency of catamenial diarrhœa; whether it occur—

1. At the prodroma of menstruation.
2. During its regular establishment.
3. At its cessation.

1st. As a symptom of the prodroma of menstruation diarrhœa rarely occurs; according to Dr. Tilt's observations, once only in 161 cases.

2d. As a symptom of regularly established menstruation, it occurred in 88 instances of 161 women carefully interrogated as to this point. In these 88 cases it *preceded* the menstrual flow in 45; *accompanied* it in 31; *both preceded and accompanied* it in 10; and in 2 instances habitually *followed* it. In the cases of precursory diarrhœa the bowels were in general costive until the cessation of the catamenia.

3d. As a symptom of menstruation at its cessation diarrhœa was much less frequent than is generally believed, occurring in 1 per cent. only of such cases.

The diarrhœa is generally unattended by pain, but sometimes preceded for two or three days by nausea and slight colics; in one case these symptoms lasted eight days previously to the diarrhœa. When it occurs at the change of life, it generally appears at irregular intervals. It may, however, adopt the irregularity of the catamenia. As a general rule, when diarrhœa has habitually accompanied menstruation, there is, at the change of life, a gradual diminution and ultimate cessation of *both* discharges. As a prodromic symptom diarrhœa scarcely ever occurs. It is found in 8 per cent. of cessation cases, and a very frequent precursory symptom of fully established menstruation. He therefore drew the physiological conclusions, that for the performance of menstruation the ovaries not only determine their secretion from the womb, but often call into concentraneous action most of the organs which, being subservient to nutrition, are supplied by the same ganglionic system, and particularly the intestines, with which they are in such close juxtaposition. He then proceeded to the rules which he thought should be adopted in the administration of purgatives:—

1. During the prodroma.
2. During the regular establishment.
3. At the cessation of menstruation.

1st. *Use of purgatives during the prodroma.*—Dr. Tilt concluded from the rarity of this function being preceded by diarrhœa, that nature does not indicate purgatives at this period, while experience has proved the painful consequences frequently entailed by their being administered as emmenagogues.

2d. *Use of purgatives during fully established menstruation.*—In this stage, Dr. Tilt considers they may be given with advantage in serious organic or uterine lesion contra-indicating their use, the great point being so to give them as not to interfere with the menstrual type. As this cannot be done with impunity in the majority of women, brisk purgatives given a few days before the menstrual epoch often anticipate it by several days, and thus vex nature in one of her most constant laws.

Previously, therefore, to their exhibition, an inquiry should be made as to the number of days the diarrhœa usually precedes the menstruation; or, if the patient has not the symptom, the date of appearance of other menstrual symptoms will be a sure guide as to the fit time for giving purgatives.

Dr. Tilt next indicated the value of purgatives in amenorrhœa and chlorosis. He does not confide in them alone, but they only form the initial part of the treatment. He first gives an ameto-cathartic, and then steel and bitters. If the appetite does not improve, and the bowels remain sluggish, he omits the tonic treatment, and gives another emeto-cathartic.

Dr. Tilt is of opinion that purgatives given just before the menstrual epoch are injudicious, as they might increase the flow nature seeks to diminish, but recommends the frequent use of mild aperients to diminish the plethora of the abdominal viscera. Purgatives should also be given after the *expiration* of menstruation, with the same view.

Dr. Tilt frequently orders five or ten grains of the soap and aloes pill before dinner, and states that he has never seen hæmorrhoidal affections caused by the use of this pill, but often relieved. He also often administers flowers of sulphur alone, or else with an admixture of sesquicarbonate or biborate of soda, in the proportion of ʒj. to ℥j., and sometimes with the addition of 5 to 10 grains of ipecacuanha: of this powder, the dose is ℈j. ad ℈ji. ounce a day in milk.

In the discussion that followed, the connection of diarrhœa with menstruation was shown not to be an entirely novel observation. Some of the speakers considered it to be indicative of a morbid condition of the uterus, and others as a revulsive to the uterine secretion. Dr. Tilt's opinions were supported by few of the Fellows who spoke on the question.

It was also argued that the number of observations was too small to enable the author of the paper to discover a natural law, one Fellow (Dr. King,) asserting that statists had agreed that at least ten thousand observations of a vital phenomenon were required before the law regulating such vital phenomenon could be safely deduced.

It was stated that Dr. Butler Lane had published a paper proving, from 200 observations, that *constipation* was the attendant upon menstruation.

Objections were also offered to the exhibition of the aloes, as being productive of hæmorrhoids.

Dr. Tilt, in reply, said that he brought forward the results he had already obtained with the full knowledge of their imperfect form, in the hope of testing their value by the experience of others. He had learned with pleasure that Dr. Butler Lane had deemed the subject worthy of investigation, and he owned that he had derived information from the present discussion; while he regretted that he had not met several objections by stating in his paper the manner in which he had conducted the inquiry. His statements did not refer to *diseased*, but truthfully represented the phenomena of *healthy* menstruation, and could stand the physiological deductions he had drawn from them; for he had long been in the habit of inquiring from all the female patients who sought his advice, what was the habitual state of the menstrual function during the period of health. Chlorotic patients, whom he invariably found subject to constipations or those laboring from severe uterine disease (in whom as Dr. Bennett had stated, diarrhœa existed, and often assumed a morbid intensity proportioned to that of the disease,) were carefully excluded from the 161 cases. Such facts, as well as the judicious observations of Dr. Murphy and Dr. Hunt, confirmed Dr. Tilt in his views. He regretted the impossibility of reconciling the discrepancies of those who had spoken the properties of aloes, being himself in the constant habit of prescribing the aloes and soap pill at dinner-time as an eccaprotic. He had never seen it produce hæmorrhoidal affections; and he maintained, that those whose opinions were at variance with his own must have given it in a different manner, in larger quantities, and to patients predisposed to such affection.—*London Med. Gaz.*, March 7, 1851.

